# The association between depression and esophageal cancer in China: a multicentre population-based study

**DOI:** 10.1186/s12888-021-03534-2

**Published:** 2021-11-10

**Authors:** Juan Zhu, Shanrui Ma, Yueyue Zhou, Ru Chen, Shuanghua Xie, Zhengkui Liu, Xinqing Li, Wenqiang Wei

**Affiliations:** 1 National Central Cancer Registry, National Cancer Center / National Clinical Research Center for Cancer / Cancer Hospital, Chinese Academy of Medical Sciences and Peking Union Medical College, Beijing, 100021 China; 2 Key Laboratory of Mental Health, Institute of Psychology, Chinese Academy of Sciences, Beijing, 100101 China

**Keywords:** Esophageal cancer, Depression, PHQ-9, Endoscopy, High-risk regions

## Abstract

**Background:**

Esophageal cancer (EC) is one of the leading contributors to the global burden of cancer, and the underlying mechanism is still unknown. Recently, there has been a growing interest in understanding modifiable psychosocial risk factors, particularly depression, to prevent EC and reduce morbidity and mortality. However, related research is sparse and has been ignored. The study was designed to assess the association between depression and EC in China.

**Methods:**

From 2017 to 2019, a population-based multicenter study was conducted in high-risk regions of EC. Participants underwent a free endoscopy screening. If the endoscopic results were suspicious, a pathological biopsy was applied to confirm. Depression was measured with Patient Health Questionnaire-9 (PHQ-9). In addition, information on demographic characteristics and risk factors was collected from participants by trained interviewers using uniform questionnaires.

**Results:**

After Endoscopy and pathologic diagnosis, 15,936 participants in high-risk regions of EC (ECHRRs) were enrolled, 10,907 (68.44%) of which were diagnosed health, 4048 (25.40%) with esophagitis, 769 (4.83%) with low-grade intraepithelial neoplasia (LGIN), 157 (0.99%) with high-grade intraepithelial neoplasia (HGIN), and 55 (0.35%) with EC, respectively. The overall prevalence of depression symptoms of participants was 4.16% (health: 4.63%, esophagitis: 2.99%, LGIN: 2.99%, HGIN: 5.73%, and EC: 9.09%). Multiple logistic regression analyses revealed that the unadjusted OR (95% CI) between depression and each esophageal pathology grades were esophagitis 0.93 (0.92-0.95), LGIN 0.97 (0.94-0.99), HGIN 1.05 (1.00-1.10), and EC 1.04 (0.97-1.14), respectively. However, after adjustment for potential confounders (age, gender, region, alcohol consumption, BMI), no statistically significant associations between depression and EC (adjusted OR = 1.10, 0.99-1.21) and esophageal lesions (esophagitis: adjusted OR = 1.02, 0.99-1.04; LGIN: adjusted OR = 0.98, 0.95-1.01; HGIN: adjusted OR = 1.04, 0.98-1.11) were observed in this study.

**Conclusions:**

No significant association was observed between depression and EC in the study. Future prospective cohort studies are needed to verify this preliminary finding.

## Background

Esophageal cancer (EC) is one of the most prevalent malignancies with high mortality and increasing incidence [1, 2]. In 2015, the incidence and mortality of EC in China were 17.9 per 100,000 and 13.7 per 100,000 [3, 4]. One-half of new cases occur in China, imposing a heavy economic burden and mental stress on families and society [3–5]. Like most common malignant tumors, EC is a complex disease with multifactorial etiology. Both genetic and environmental factors influence the risk of developing the disease [6–10]. In the past, most etiological studies of EC focused on biology, and social or psychological factors were easily ignored [11–14].

Growing evidence has shown that depression may exert an etiologic role in cancer [15–19]. A recent meta-analysis of 51 prospective studies showed that depression and anxiety disorders could cause a significant 13% increase in cancer risk and a 21% increase in cancer-specific mortality [20]. Evidence associated with depression and cancer indicated an increased cancer risk in individuals with depression [21–23]. However, many previous studies on depression and cancer have primarily focused on breast, lung, colorectal cancers [21, 22]. Only a few studies have explored the relationship between depression and EC-specific risk. A meta-analysis reviewing depression and anxiety concerning cancer incidence and mortality covered 21 common tumors [20], but a recent meta-analysis found that only one study concerned depression and the risk of esophageal cancer [24]. The evidence of which was still insufficient. Therefore, a population-based, multicenter study was implemented to estimate the status of depression in high-risk regions of the EC (ECHRRs) and evaluate the association between depression and EC, aimed to provide clues for preliminary screening and prevention of EC and fill the gap in this field.

## Methods

### Study design

**Fig. 1:**
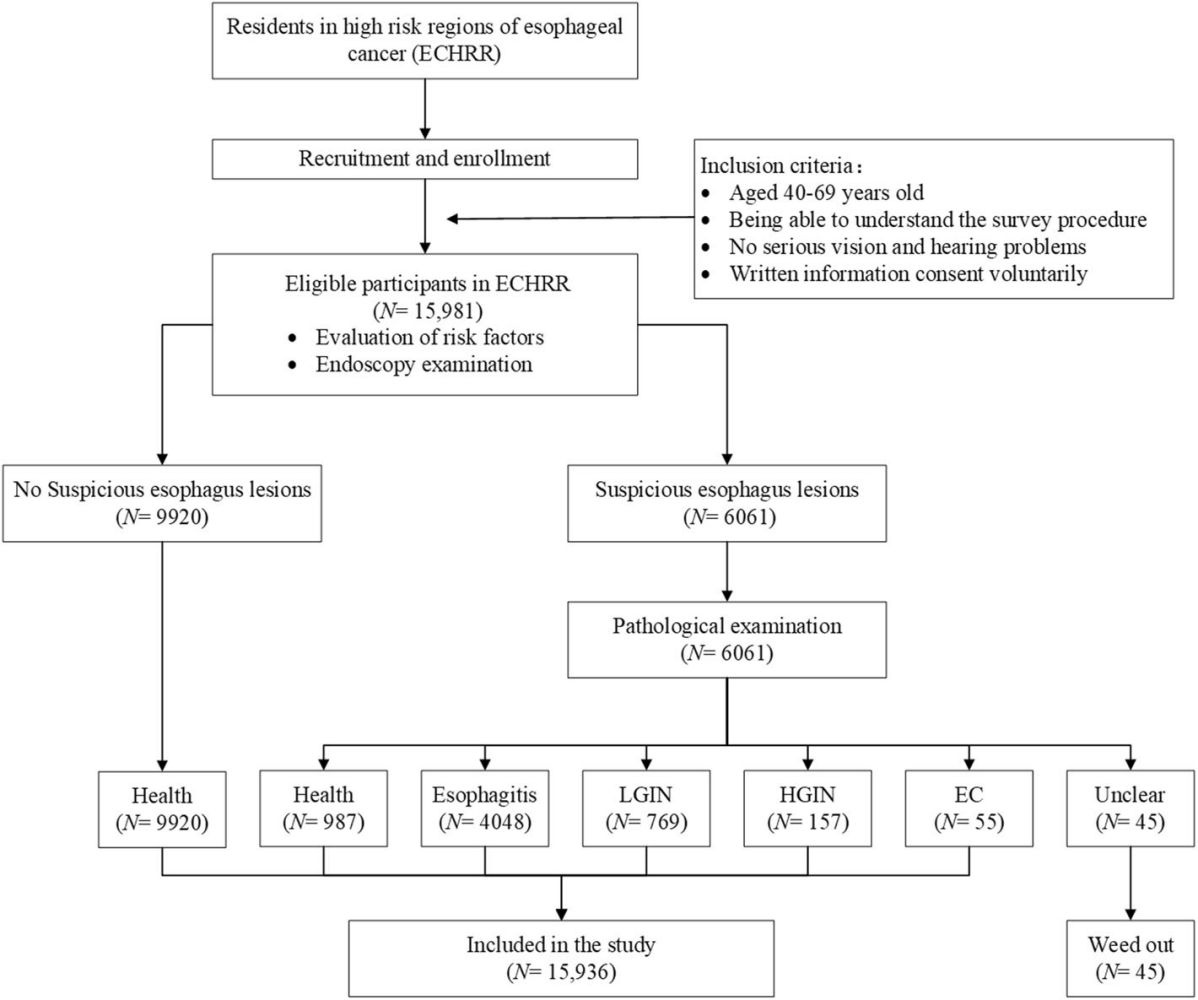
The flowchart of the study. Training staff interviewed eligible participants via a uniform and laptop-based questionnaire to collect information on their exposure to risk factors (psychological information included) (*N* = 15,981). An esophageal endoscopy examination would be performed after the questionnaire (*N* = 15,981). If esophageal endoscopy results were suspicious (*N* = 6061), a pathological biopsy would be further conducted (*N* = 6061). Screeners were diagnosed as health (*N* = 987), esophagitis (*N* = 4048), low-grade intraepithelial neoplasia (LGIN) (*N* = 769), high-grade intraepithelial neoplasia (HGIN) (*N* = 157), esophageal cancer (EC) (*N* = 55), and unclear (*N* = 45)

The national cohort of esophageal cancer (NCEC) is a multi-center prospective cohort study of EC and precancerous lesions based on high-risk populations in China [25]. Details on the cohort have already been published [25]. This study is based on the NCEC cohort and provided free gastroscopy screening services for upper gastrointestinal tumors to residents aged 40 to 69 years in five ECHRRs (Linzhou, Henan; Cixian, Hebei; Feicheng, Shandong; Yangzhong, Jiangsu; Yanting, Sichuan) from May 2017 to November 2019 (Fig. 1). All participants were recruited and interviewed face-to-face by trained staff. A uniform questionnaire was used to collect their basic information, including living and eating habits, disease history, family tumor history, and other exposure factors. Then, eligible participants received an upper gastrointestinal endoscopy examination. If an endoscopy detects suspicious esophageal lesions, esophageal pathology would be applied to confirm clinical health, esophagitis, low-grade intraepithelial neoplasia (LGIN), high-grade intraepithelial neoplasia (HGIN), and EC. The details related to the design of the NCEC are described on the website (http://www.ncec-China.cn/cmmct.html) and elsewhere [25].

### Study participants

The inclusion criteria included: (a) residents aged 40-69 years old; (b) no severe vision or hearing problems; (c) be able to comprehend the survey process properly; and (d) being competent to provide written informed consent. The exclusion criteria included: (a) previous diagnosis of EC or other cancers and (b) being contraindications for endoscopic examinations (e.g., acute perforation of the upper digestive tract, severe heart, lung, kidney, brain dysfunction, and multi-organ failure).

### Instruments

Before the esophageal endoscopy examination, all depression surveys were conducted using the Chinese version of the Patient Health Questionnaire-9 (PHQ-9). The PHQ-9 is a self-reported questionnaire assessing the presence and severity of depression with good test-retest reliability and good validity in primary medical care and clinical practice [26, 27].

PHQ-9 consists of 9 items (anhedonia, depressed mood, sleep problems, fatigue, weight/appetite change, feelings of worthlessness/guilt, poor concentration, psychomotor retardation/agitation, thoughts of self-harm/suicidal ideations) that are based directly on the nine diagnostic criteria for major depression in the Diagnostic and Statistical Manual of Mental Disorders, Fifth Edition (DSM-V) [28]. PHQ-9 has 4 response scores representing the frequency of each depressive symptom in the past 2 weeks (0 = “not at all”, 1 = “several days”, 2 = “more than half the days”, and 3 = “nearly every day”). The depression score was the total of each item from 0 to 27. A higher depression score indicates severe depression. Depression score is also used as a binary variable: cutoff score at 7, meaning 7 or above is depressive [29].

### Covariate variables

Based on the results of face-to-face interviews and a comprehensive literature review. The following covariates were included in the study: age (≤50y, 51-60y, >60y), gender, region, alcohol consumption [30, 31], and body mass index (BMI) [32–36]. Alcohol consumption was used to evaluate the frequency of drinking in the past year, divided into never (or almost never), only occasionally, most days (or almost every day). Additionally, both weight and height were measured on the endoscopy examination day, BMI was calculated as the weight in kilograms divided by the square of the height in meters (kg/m^2^), and classified according to the World Health Organization (WHO) guidelines: underweight: < 18.5, normal ≥18.5 and < 25, overweight ≥25 and < 30, and obese ≥30.

### Statistics

SAS version is 9.4 statistical programs (SAS Institute, Cary, NC) was used for data management and analyses. The continuous and qualitative variables were summarized as mean ± standard deviation (SD) or median with the first and third quartile (Q1-Q3) and frequency (%), respectively. We used the Student’s t-test for normally distributed variables and Kruskal-Wallis for non-parametric variables to analyze the continuous data. Categorical variables were compared using Chi-square or Fisher’s exact test. Multiple linear regression models (stepwise) were performed to test for correlation between covariates before covariate modeling. Collinearity diagnostics using the variance inflation factor (VIF) showed no evidence of collinearity among covariates (VIF < 10.0) [37]. Finally, we performed multivariable-adjusted logistic regressions to determine the association between depression (PHQ-9 scores) and different esophageal lesions. Specifically, model 1 was non-adjusted that estimated the raw contribution of depression to the prediction of esophageal lesions; model 2 was adjusted for age, gender, and region; model 3 was adjusted for age, gender, region, alcohol, and BMI. All tests of significance were two-tailed, and *P* < 0.05 was examined statistically significant.

### Ethics statement

The study was performed in accordance with the institutional research ethics guidelines and the Helsinki declaration. Formal ethics approval was granted by the Ethics Committee of Cancer Institute and Hospital, Chinese Academy of Medical Sciences (No.16-171/1250). All the participants were informed about the purpose of the study and provided written informed consent.

## Results

### The screening detection and diagnosis of different esophageal lesions

As showed in Fig. 1, A total of 15,981 eligible participants were enrolled in five ECHRRs. After endoscopy and pathologic diagnosis, 15,936 participants were entered in the present study, as 45 participants had an unclear pathological diagnosis. And 10,907 health (68.44%), 4048 esophagitis (25.40%), 769 LGIN (4.83%), 157 HGIN (0.99%) and 55 EC (0.35%) cases were confirmed, respectively.

### Baseline characteristics of participants with different esophageal lesions

The baseline characteristics of participants are shown in Table 1. The mean age of all participants was (55.44 ± 7.74) years. Healthy people are younger than others (*P* < 0.001). Over half of the participants (58.56%) were women, while the HGIN and EC group included more males patients (50.32, 56.36%). Moreover, the differences in baseline characteristics between the healthy group and the other esophageal lesions groups were statistically significant in terms of region, marital status, highest education level, occupation, household income, smoking status, alcohol and tea consumption, physical activities, life satisfaction status, self-rated health status, and BMI (all *P* < 0.05).

**Table 1:** Baseline characteristics of participants with different esophageal lesions in ECHRRs

Characteristics	Esophageal pathology						Chi-square test	*P* Value
	Health *N* = 10,907	Esophagitis *N* = 4048	LGIN *N* = 769	HGIN *N* = 157	EC *N* = 55	Total *N* = 15,936		
**Age, (Mean ± SD)**	54.48 ± 7.66	56.65 ± 7.53	60.65 ± 6.23	62.71 ± 5.86	61.49 ± 6.42	55.44 ± 7.74	466.28^a^	< 0.001
**PHQ-9 score, Median (Q1-Q3)**	1.00 (0.00–4.00)	0.00 (0.00–4.00)	0.00 (0.00–4.00)	1.00 (0.00–4.00)	1.00 (0.00–4.00)	0.00 (0.00–4.00)	159.74^b^	< 0.001
**Age (years), n (%)**							249.37	< 0.001
≤ 50	3776 (34.62)	963 (23.79)	48 (6.24)	5 (3.18)	3 (5.45)	4795 (30.09)		
51–60	4249 (38.96)	1636 (40.42)	292 (37.97)	40 (25.48)	17 (30.91)	6234 (39.12)		
> 60	2882 (26.42)	1449 (35.81)	429 (55.79)	112 (71.34)	35 (63.64)	4907 (30.79)		
**Gender, n (%)**							12.74	0.013
Male	4471 (40.99)	1686 (41.65)	337 (43.82)	79 (50.32)	31 (56.36)	6604 (41.44)		
Female	6436 (59.01)	2362 (58.35)	432 (56.18)	78 (49.68)	24 (43.64)	9332 (58.56)		
**Region, n (%)**							186.87	< 0.001
Linzhou	1260 (11.55)	767 (18.95)	170 (22.11)	38 (24.2)	13 (23.64)	2248 (14.11)		
Cixian	2878 (26.39)	1041 (25.72)	203 (26.4)	44 (28.03)	15 (27.27)	4181 (26.24)		
Feicheng	3222 (29.54)	375 (9.26)	212 (27.57)	35 (22.29)	19 (34.55)	3863 (24.24)		
Yanting	3193 (29.27)	146 (3.61)	77 (10.01)	32 (20.38)	6 (10.91)	3454 (21.67)		
Yangzhong	354 (3.25)	1719 (42.47)	107 (13.91)	8 (5.1)	2 (3.64)	2190 (13.74)		
**Marital status, n (%)**							76.66	< 0.001
Married	10,294 (94.38)	3682 (90.96)	689 (89.60)	137 (87.26)	49 (89.09)	14,851 (93.19)		
Unmarried/Living alone/divorced/widowed	613 (5.62)	366 (9.04)	80 (10.40)	20 (12.74)	6 (10.91)	1085 (6.81)		
**Highest education level, n (%)**							86.02	< 0.001
Primary school or below	5393 (49.45)	1845 (45.58)	454 (59.04)	104 (66.24)	35 (63.64)	7831 (49.14)		
Junior or senior high school	5424 (49.73)	2152 (53.16)	313 (40.7)	53 (33.76)	20 (36.36)	7962 (49.96)		
Undergraduate or over	83 (0.76)	44 (1.09)	2 (0.26)	0 (0.00)	0 (0.00)	129 (0.81)		
Unknown	7 (0.06)	7 (0.17)	0 (0.00)	0 (0.00)	0 (0.00)	14 (0.09)		
**Occupation, n (%)**							761.90	< 0.001
Agriculture or related workers	7140 (65.46)	1745 (43.11)	495 (64.37)	121 (77.07)	46 (83.64)	9547 (59.91)		
Factory workers	1393 (12.77)	947 (23.39)	76 (9.88)	9 (5.73)	2 (3.64)	2427 (15.23)		
Housewife or househusband	1296 (11.88)	646 (15.96)	128 (16.64)	16 (10.19)	3 (5.45)	2089 (13.11)		
Administrator or manager or professional or technical	216 (1.98)	114 (2.82)	13 (1.69)	2 (1.27)	1 (1.82)	346 (2.17)		
Self-employed or sales or service workers	552 (5.06)	309 (7.63)	20 (2.60)	2 (1.27)	1 (1.82)	884 (5.55)		
Retired	97 (0.89)	115 (2.84)	9 (1.17)	4 (2.55)	0 (0.00)	225 (1.41)		
Unemployed	42 (0.39)	29 (0.72)	4 (0.52)	1 (0.66)	0 (0.00)	76 (0.48)		
Unknown	171 (1.57)	143 (3.53)	24 (3.12)	2 (1.27)	2 (3.64)	342 (2.15)		
**Household income (ten thousand RMB/year), n (%)**							1147.08	< 0.001
< 3.0	1591 (14.59)	631 (15.59)	211 (27.44)	48 (30.57)	11 (20)	2492 (15.64)		
3.0–7.0	6617 (60.67)	1579 (39.01)	367 (47.72)	78 (49.68)	33 (60)	8674 (54.43)		
7.0–11.0	2247 (20.6)	1046 (25.84)	142 (18.47)	26 (16.56)	11 (20)	3472 (21.79)		
≥ 11.0	452 (4.14)	792 (19.57)	49 (6.37)	5 (3.18)	0 (0.00)	1298 (8.15)		
**Smoking status, n (%)**							22.80	0.004
Do not smoke	8640 (79.22)	3099 (76.56)	602 (78.28)	117 (74.52)	36 (65.45)	12,494 (78.40)		
Only occasionally	331 (3.03)	136 (3.36)	17 (2.21)	6 (3.82)	1 (1.82)	491 (3.08)		
Most days/almost every day	1936 (17.75)	813 (20.08)	150 (19.51)	34 (21.66)	18 (32.73)	2951 (18.52)		
**Alcohol consumption, n (%)**							57.13	< 0.001
Never or almost never	6922 (63.46)	2614 (64.58)	510 (66.32)	91 (57.96)	27 (49.09)	10,164 (63.78)		
Only occasionally	2535 (23.24)	806 (19.91)	135 (17.56)	31 (19.75)	10 (18.18)	3517 (22.07)		
Most days/almost every day	1450 (13.29)	628 (15.51)	124 (16.12)	35 (22.29)	18 (32.73)	2255 (14.15)		
**Tea consumption, n (%)**							414.12	< 0.001
Never or almost never	5190 (47.58)	2641 (65.24)	440 (57.22)	88 (56.05)	27 (49.09)	8386 (52.62)		
Only occasionally	2564 (23.51)	694 (17.14)	111 (14.43)	35 (22.29)	8 (14.55)	3412 (21.41)		
Most days/almost every day	3153 (28.91)	713 (17.61)	218 (28.35)	34 (21.66)	20 (36.36)	4138 (25.97)		
**Physical activities, n (%)**							132.70	< 0.001
Never or almost never	9544 (87.5)	3344 (82.61)	665 (86.48)	137 (87.26)	49 (89.09)	13,739 (86.21)		
1–2 times / week	217 (1.99)	160 (3.95)	14 (1.82)	2 (1.27)	1 (1.82)	394 (2.47)		
3–5 times / week	170 (1.56)	163 (4.03)	10 (1.30)	1 (0.64)	1 (1.82)	345 (2.16)		
Daily/almost every day	976 (8.95)	381 (9.41)	80 (10.40)	17 (10.83)	4 (7.27)	1458 (9.15)		
**Life satisfaction status, n (%)**							36.46	< 0.001
Very satisfied	2307 (21.15)	774 (19.12)	181 (23.54)	34 (21.66)	10 (18.18)	3306 (20.75)		
Basically satisfied	7622 (69.88)	2817 (69.59)	503 (65.41)	112 (71.34)	38 (69.09)	11,092 (69.60)		
General	948 (8.69)	432 (10.67)	82 (10.66)	10 (6.37)	7 (12.73)	1479 (9.28)		
Not satisfied	30 (0.28)	25 (0.62)	3 (0.39)	1 (0.64)	0 (0.00)	59 (0.37)		
**Self-rated health status, n (%)**							254.46	< 0.001
Excellent	2592 (23.76)	739 (18.26)	191 (24.84)	36 (22.93)	14 (25.45)	3572 (22.41)		
Good	6449 (59.13)	2169 (53.58)	382 (49.67)	82 (52.23)	26 (47.27)	9108 (57.15)		
General	1766 (16.19)	1092 (26.98)	188 (24.45)	36 (22.93)	15 (27.27)	3097 (19.43)		
Fair-poor	100 (0.92)	48 (1.19)	8 (1.04)	3 (1.91)	0 (0.00)	159 (1.00)		
**BMI (kg/m**^**2**^**), n (%)**							31.67	0.002
< 18.5	187 (1.71)	79 (1.95)	18 (2.34)	5 (3.18)	1 (1.82)	290 (1.82)		
18.5–25.0	6107 (55.99)	2364 (58.40)	458 (59.56)	102 (64.97)	36 (65.45)	9067 (56.90)		
25.0–30.0	3900 (35.76)	1402 (34.63)	253 (32.90)	44 (28.03)	17 (30.91)	5616 (35.24)		
≥ 30.0	713 (6.54)	203 (5.01)	40 (5.20)	6 (3.82)	1 (1.82)	963 (6.04)		
**Depression symptoms**							27.54	< 0.001
Depression (PHQ-9 ≥ 7)	505 (4.63)	121 (2.99)	23 (2.99)	9 (5.73)	5 (9.09)	663 (4.16)		
Non-depression (PHQ-9 < 7)	10,402 (95.37)	3927 (97.01)	746 (97.01)	148 (94.27)	50 (90.91)	15,273 (95.84)		

*SD* Standard deviation, *RMB* Renminbi, *BMI* Body mass index, *LGIN* Low-grade intraepithelial neoplasia, *HGIN* High-grade intraepithelial neoplasia, *EC* Esophageal cancer

Q1: First Quartile, the 25th percentile; Q3: Third Quartile, the 75th percentile

a Age between groups was compared using analysis of variance (ANOVA)

b Kruskal-Wallis test

### The depression symptoms of participants with esophageal lesions

As showed in Table 2, the overall prevalence of depression (PHQ-9 > 7) in the study was 4.16% (663/15,936). The corresponding prevalence of depression of participants diagnosed with health, esophagitis, LGIN, HGIN, and EC were 4.63% (505/10,907), 2.99% (121/4048), 2.99% (23/769), 5.73% (9/157), and 9.09% (5/55), respectively (*P* < 0.001). The significant variations in depression among the different demographic characteristics and life habits such as region (*P* < 0.001), the highest education level (*P* = 0.021), occupation (*P* < 0.001), household income (*P* = 0.006), alcohol consumption (*P* < 0.001), tea consumption (*P* < 0.001), physical activities (*P* < 0.001), life satisfaction status (*P* < 0.001), self-rated health status (*P* < 0.001), and BMI (*P* = 0.010).

**Table 2:** Baseline characteristics of participants with depression symptoms in ECHRRs

Characteristics	Depression		Non-depression		Total		Chi-square test	*P* Value
	***n*** = 663	%	***n*** = 15,273	%	***n*** = 15,936	%		
**Age (Mean ± SD)**	55.45 ± 7.51		55.44 ± 7.75		55.44 ± 7.74		-0.047^a^	0.963
**Age (years)**							0.29	0.867
≤ 50	194	29.26	4601	30.13	4795	30.09		
51–60	265	39.97	5969	39.08	6234	39.12		
> 60	204	30.77	4703	30.79	4907	30.79		
**Gender**							0.30	0.587
Male	268	40.42	6336	41.48	6604	41.44		
Female	395	59.58	8937	58.52	9332	58.56		
**Region**							181.80	< 0.001
Linzhou	70	10.56	2178	14.26	2248	14.11		
Cixian	217	32.73	3964	25.95	4181	26.24		
Feicheng	94	14.18	3769	24.68	3863	24.24		
Yanting	253	38.16	3201	20.96	3454	21.67		
Yangzhong	29	4.37	2161	14.15	2190	13.74		
**Marital status**							1.64	0.200
Married	626	94.42	14,225	93.14	14,851	93.19		
Unmarried/Living alone/divorced/widowed	37	5.58	1048	6.86	1085	6.81		
**Highest education level**							9.78	0.021
Primary school or below	350	52.79	7481	48.98	7831	49.14		
Junior or senior high school	304	45.85	7658	50.14	7962	49.96		
Undergraduate or over	6	0.90	123	0.81	129	0.81		
Unknown	3	0.45	11	0.07	14	0.09		
**Occupation**							111.59	< 0.001
Agriculture or related workers	519	78.28	9028	59.11	9547	59.91		
Factory workers	44	6.64	2383	15.60	2427	15.23		
Housewife or househusband	56	8.45	2033	13.31	2089	13.11		
Administrator or manager or professional or technical	12	1.81	334	2.19	346	2.17		
Self-employed or sales or service workers	17	2.56	867	5.68	884	5.55		
Retired	5	0.75	220	1.44	225	1.41		
Unemployed	2	0.30	74	0.48	76	0.48		
Unknown	8	1.21	334	2.19	342	2.15		
**Household income (ten thousand RMB/year)**							20.99	0.006
< 3.0	85	12.82	2407	15.76	2492	15.64		
3.0–7.0	412	62.14	8262	54.10	8674	54.43		
7.0–11.0	132	19.91	3340	21.87	3472	21.79		
≥ 11.0	34	5.13	1264	8.28	1298	8.15		
**Smoking status**							0.88	0.644
Do not smoke	529	79.79	11,965	78.34	12,494	78.40		
Only occasionally	18	2.71	473	3.10	491	3.08		
Most days or almost every day	116	17.50	2835	18.56	2951	18.52		
**Alcohol consumption**							57.81	< 0.001
Never or almost never	415	62.59	9749	63.83	10,164	63.78		
Only occasionally	205	30.92	3312	21.69	3517	22.07		
Most days or almost every day	43	6.49	2212	14.48	2255	14.15		
**Tea consumption**							52.02	< 0.001
Never or almost never	325	49.02	8061	52.78	8386	52.62		
Only occasionally	215	32.43	3197	20.93	3412	21.41		
Most days or almost every day	123	18.55	4015	26.29	4138	25.97		
**Physical activities**							18.55	< 0.001
Never or almost never	606	91.40	13,133	85.99	13,739	86.21		
1–2 times / week	9	1.36	385	2.52	394	2.47		
3–5 times / week	7	1.06	338	2.21	345	2.16		
Daily or almost every day	41	6.18	1417	9.28	1458	9.15		
**Life satisfaction status**							65.40	< 0.001
Very satisfied	73	11.01	3233	21.17	3306	20.75		
Basically satisfied	482	72.70	10,610	69.47	11,092	69.60		
General	103	15.54	1376	9.01	1479	9.28		
Not satisfied	5	0.75	54	0.35	59	0.37		
**Self-rated health status**							36.62	< 0.001
Excellent	105	15.84	3467	22.70	3572	22.41		
Good	434	65.46	8674	56.79	9108	57.15		
General	108	16.29	2989	19.57	3097	19.43		
Fair-poor	16	2.41	143	0.94	159	1.00		
**BMI (kg/m2)**							11.34	0.010
< 18.5	14	2.11	276	1.81	290	1.82		
18.5–25.0	409	61.69	8658	56.69	9067	56.90		
25.0–30.0	194	29.26	5422	35.50	5616	35.24		
≥ 30.0	46	6.94	917	6.00	963	6.04		
**Esophageal pathology**							27.54	< 0.001
Health	505	76.17	10,402	68.11	10,907	68.44		
Esophagitis	121	18.25	3927	25.71	4048	25.40		
LGIN	23	3.47	746	4.88	769	4.83		
HGIN	9	1.36	148	0.97	157	0.99		
EC	5	0.75	50	0.33	55	0.35		

*SD* Standard deviation, *RMB* Renminbi, *BMI* Body mass index, *LGIN* Low-grade intraepithelial neoplasia, *HGIN* High-grade intraepithelial neoplasia, *EC* Esophageal cancer

a t test

### Multiple linear regression analysis of depression (PHQ-9 scores) and baseline characteristics

Table 3 shows the regression equation results by multiple linear regression analysis (forward stepwise selection method). Depression (PHQ-9 score as dependent variable) is related to occupation, life satisfaction status, region, household income, physical activities, self-rated health status, tea consumption, BMI, alcohol consumption, marital status, highest education level. The results of collinearity diagnostics showed that all VIF values were below 10, which indicated that no severe multicollinearities exist between the independent variables in this study.

**Table 3:** Multiple linear regression analysis of depression (PHQ-9 score) and baseline characteristics ^a^

Characteristics	Unstandardized β	Coefficients Std.Error	Standardized β	95% CI for β	*P value*	Collinerity Statistics	
						Tolerance	VIF
Occupation	0.191	0.008	0.199	(0.176, 0.205)	< 0.001	0.845	1.184
Life satisfaction status	−0.151	0.008	−0.150	(−0.167, −0.135)	< 0.001	0.794	1.260
Region	−0.331	0.018	−0.148	(−0.366, −0.297)	< 0.001	0.852	1.173
Household income	−0.111	0.009	−0.096	(−0.128, −0.094)	< 0.001	0.934	1.071
Physical activities	0.130	0.011	0.088	(0.108, 0.152)	< 0.001	0.904	1.106
Self-rated health status	−0.073	0.008	−0.073	(− 0.088, − 0.057)	< 0.001	0.798	1.253
Tea consumption	0.027	0.007	0.031	(0.013, 0.041)	< 0.001	0.781	1.280
BMI	−0.139	0.032	− 0.031	(− 0.203, − 0.075)	< 0.001	0.982	1.018
Alcohol consumption	0.022	0.007	0.025	(0.008, 0.036)	0.002	0.825	1.213
Marital status	−0.030	0.012	−0.018	(− 0.053, − 0.006)	0.013	0.962	1.040
Highest education level	0.012	0.006	0.015	(0.000, 0.024)	0.048	0.928	1.078

a Adjusted R^2^ = 0.164

Dependent Variable: PHQ-9 score

### The association between depression and the esophageal lesions

Afterward, we performed a multiple logistics regression analysis to explore the relationships between depression (PHQ-9 scores) and different esophageal lesions *(*Table 4*)*. Compared with healthy participants (reference), the unadjusted OR (95% CIs) between depression and each grade of esophageal pathology were 0.93 (0.92-0.95), 0.97 (0.94-0.99), 1.05 (1.00-1.10), and 1.04 (0.97-1.14), respectively. After further adjustment for the age, gender, region, alcohol consumption, and BMI, depression has not shown a significant association with all esophageal pathology. The corresponding OR (95% CI) of the associations were 1.02 (0.99-1.04), 0.98 (0.95-1.01), 1.04 (0.98-1.11), and 1.10 (0.99-1.21), respectively.

**Table 4:** Odds ratios (OR) of association between depression symptoms and esophageal lesions in ECHRRs

Esophageal pathology	Health	Esophagitis OR (95%CI)	LGIN OR (95%CI)	HGIN OR (95%CI)	EC OR (95%CI)
Model 1^a^	Ref	0.93 (0.92–0.95)^**^	0.97 (0.94–0.99)^**^	1.05 (1.00–1.10)^*^	1.04 (0.97–1.14)
Model 2^b^	Ref	1.01 (0.99–1.03)	0.97 (0.94–1.01)	1.04 (0.98–1.11)	1.08 (0.98–1.19)
Model 3^c^	Ref	1.02 (0.99–1.04)	0.98 (0.95–1.01)	1.04 (0.98–1.11)	1.10 (0.99–1.21)

*OR* Odds ratio, *95%CI* 95% Confidence interval, *LGIN* Low-grade intraepithelial neoplasia, *HGIN* High-grade intraepithelial neoplasia, *EC* Esophageal cancer, *Ref* Reference

a Model 1: Univariate model including depression (depression was used as a continuous variable)

b Model 2: Model 1 + age + gender + region

c Model 3: Model 2 + alcohol consumption + BMI

* *P* < 0.05; ** *P* < 0.01

## Discussion

With the transition from a biological medical model to a biopsychosocial model, the impact of psychosocial factors on cancer progression has attracted much attention. This study is the first to focus on depression symptoms and esophageal lesions in a large-scale multi-center population on a global scale, which filled the gap in this field. Findings from this population-based study supported the view that the prevalence of depression in ECHRRs was high, especially for EC. Nevertheless, we found no evidence of an association between depression and the risk of either esophageal lesions or EC.

Depression is among the most prevalent and disabling psychological disorders worldwide and affects 350 million people [38–40]. Evidence from the China Kadoorie Biobank (CKB) study of 0.5 million adults indicated that depression could not be ignored in China [41]. The latest National Health Survey (NHS) in 2019 reported that the lifetime prevalence of depressive disorders was 6.8% in China [42]. In our study, the overall prevalence of depression in ECHRRs was 4.16%, participants with EC was 9.09%, which was higher than the national level. The possible explanation is that EC is one of the most human malignant tumors, with high mortality and poor survival [2, 3], threatening the health of people living in ECHRRs. Residents have a higher risk of EC and suffer higher stress and anxiety, prioritizing those under limited psychological health resources.

Interestingly, the prevalence of depression was lower in patients with esophagitis and LGNI than health in our study. A possible reason was that the study was carried out in screening populations instead of clinical medical records, so most screening participants were healthy people. It indicated that the healthy people had strong health awareness on cancer screening, paid much attention to their body, and even worry about their health, which may lead to a high level of depressive disorders. The second explanation was that patients with esophagitis and LGIN have almost no physical discomfort and do not need special treatment and surgery, which would bring little psychological burden. Besides, patients with esophagitis and LGIN maybe feel lucky and glad that they do not have cancer.

Our study found no evidence of an association between depression and the risk of either esophageal lesions or EC. The following factors may explain the observed non-significant association. 1) There is a lack of solid evidence of a positive association between cancer and depression, and the existing epidemiological studies have yielded conflicting results. Several meta-analyses and systematic reviews have been published on the topic but have reported mixed results. More recently, a meta-analysis published in 2007 suggested a small and modestly significant relationship between depression and the risk of cancer incidence (RR 1.13; 95% CI:1.06–1.19) [20]. However, a meta-analysis found that clinically diagnosed depressive disorder people do not have an elevated risk for cancer incidence (OR 1.15; 95% CI:0.85–1.56) [43]. So far, the research on the relationship between esophageal cancer and depression is sparse, and our findings filled the gap in the field. 2) Although the total screening participants is enough, the HGNI and EC from the screening population were relatively more minor, which means that related results carry uncertainty to some extent and need to be interpreted cautiously. 3) We tried to put many covariates in modeling regression before, and the results showed a positive association between depression and esophageal cancer. On second consideration, considering that the smallest group only includes 55 people and the possibility of overfitting, we made a priori selection for the current confounding factors, and the results turned out to change from positive correlation to non-significant correlation. Despite our negative results between depression and EC-related diseases, depression must not be ignored because increasing evidence found that depression may be influencing the progression of cancer [15, 16, 44, 45].

Reverse causation may exist between depression and EC because cancer diagnosis could influence the mental health status or rise to depression [46]. Unlike most previous studies, our research evaluated the association of depression and EC in a population and excluded people with a cancer history, minimizing the influence of reverse causality. Furthermore, the results indicated that distress symptoms alone appear to be relatively less harmful to cancer development [43]. Considering that most human cancers have a long latency period and are difficult to detect during the early stages of cancer, an association between depression and EC was not identified in this cross-sectional study. In addition, lifestyle and behavioral changes may influence the association between depression and cancer indirectly. People with depression are more likely to have unhealthy lifestyle habits [47]. For instance, according to Watts, most individuals with depressive disorders abuse alcohol in search of disinhibition or reduce emotional and behavioral symptoms of depression [48]. Obesity and BMI were associated with depression [49, 50]. In this cross-sectional study, the ratio of depression differed according to regions, education level, occupation, alcohol drinking habit, and BMI level. However, risk factors of depression and EC are in part shared, further complicating causal interpretations. Among those, socioeconomic status (SES), education level may play mediating roles in the association between depression and EC. Intermediary factor analysis will be taken into account in further study.

Several limitations to this pilot study have to be acknowledged. First, due to the study’s cross-sectional design, causal inferences cannot be shown, and the long-term effects of depression on EC progression are also unavailable. Second, volunteer bias may exist. Residents were willing to participate in the endoscopy program actively because of free. Third, even though we try to control the confounding factors as much as possible, the smallest group only included 55 persons; we do not have the correct number of participants to include more covariables. Therefore, we cannot control the confounding factors completely. Finally, the results in ECHRRs may not be generalization to the general population, which should be interpreted with caution.

## Conclusion

Our study took the lead in investigating the association between depression and EC in China. Findings from this population-based study supported the view that the prevalence of depression in ECHRRs was high. There is no clear evidence that depression may be a contributing factor to EC and precancerous lesions. The results should be interpreted with caution.

### Implications

Depression, causing a significant psychological burden, has long been underestimated seriously worldwide. Confronted with a lack of awareness of the psychological health of Chinese people, and there is a considerable gap in psychological services between China and developed counties. The government in China is actively promoting improving residents’ mental health literacy to 30% by 2030. The priorities of psychological health resources should be provided to high-risk populations, such as residents in ECHRRs and people screened to HGIN and EC.

## Data Availability

The datasets for this manuscript maybe not publicly available because all our data are under the regulation of the National Cancer Center in China. Requests to access the datasets should be directed to Wenqiang Wei, weiwq@cicams.ac.cn.

## Abbreviations

EC: Esophageal cancer
ECHRRs: high-risk regions of EC
NCEC: The national cohort of esophageal cancer
LGIN: Low-grade intraepithelial neoplasia
HGIN: High-grade intraepithelial neoplasia
PHQ-9: Patient Health Questionnaire-9
HPA: Hypothalamic-pituitary-adrenal axis
CKB: China Kadoorie Biobank
WHO: World Health Organization
NHS: National Health Survey
RMB: Renminbi
BMI: Body mass index
